# Intranasal Flu Vaccine Protective against Seasonal and H5N1 Avian Influenza Infections

**DOI:** 10.1371/journal.pone.0005336

**Published:** 2009-04-29

**Authors:** Mohammed Alsharifi, Yoichi Furuya, Timothy R. Bowden, Mario Lobigs, Aulikki Koskinen, Matthias Regner, Lee Trinidad, David B. Boyle, Arno Müllbacher

**Affiliations:** 1 Division of Immunology and Genetics, The John Curtin School of Medical Research, Australian National University, Canberra, Australian Capital Territory, Australia; 2 CSIRO Livestock Industries, Australian Animal Health Laboratory, Geelong, Victoria, Australia; 3 Microbiology and Infectious Diseases, Institute of Medical and Veterinary Science, Adelaide, South Australia, Australia; New York University School of Medicine, United States of America

## Abstract

**Background:**

Influenza A (flu) virus causes significant morbidity and mortality worldwide, and current vaccines require annual updating to protect against the rapidly arising antigenic variations due to antigenic shift and drift. In fact, current subunit or split flu vaccines rely exclusively on antibody responses for protection and do not induce cytotoxic T (Tc) cell responses, which are broadly cross-reactive between virus strains. We have previously reported that γ-ray inactivated flu virus can induce cross-reactive Tc cell responses.

**Methodology/Principal Finding:**

Here, we report that intranasal administration of purified γ-ray inactivated human influenza A virus preparations (γ-Flu) effectively induces heterotypic and cross-protective immunity. A single intranasal administration of γ-A/PR8[H1N1] protects mice against lethal H5N1 and other heterotypic infections.

**Conclusions/Significance:**

Intranasal γ-Flu represents a unique approach for a cross-protective vaccine against both seasonal as well as possible future pandemic influenza A virus infections.

## Introduction

The selection of virus strains for the formulation of current flu vaccines is entirely based on “educated guesses” by comparing recent virus isolates to known circulating human flu strains. Prediction of flu strains that may cause infection in any flu season (not pandemics) cannot accommodate the expected antigenic variability arising due to virus mutations (genetic drift). For example, the World Health Organization recommended the use of A/Wisconsin/67/2005[H3N2]-like virus as part of the trivalent inactivated vaccine for the Northern Hemisphere flu season 2007–2008 [1]. Yet, according to the Centers for Disease Control and Prevention, 65% of H3N2 influenza infections during the 2007–2008 flu season in the US were caused by A/Brisbane/10/2007[H3N2]-like viruses that evolved from A/Wisconsin/67/2005[H3N2]-like virus and turned out to be antigenically distinct virus that consequently rendered the vaccine ineffective [2]. This illustrates the need for a new vaccine concept that maintains high protective efficacy regardless of the antigenic variations that arise frequently due to antigenic drift.

Chemically and UV inactivated influenza virus preparations rely exclusively on antibody responses for protection and do not induce cytotoxic T (Tc) cell responses [3]. The Tc cell response to influenza is broadly cross-reactive between virus strains and is important in the recovery from primary infections [4]. We have previously reported that γ-Flu preparations can induce cross-reactive Tc cell responses [5]. Gamma-irradiation is the preferred method of inactivation of highly infectious agents for biochemical analysis, including Ebola, Marburg and Lassa viruses [6], [7], [8]. It inactivates virus infectivity by generating strand-breaks in the genetic material and has the further advantage, compared with chemical agents, of high penetration into and through biological materials [7]. In contrast to chemical treatment with formalin or β-propiolactone (currently used in the production of inactivated influenza virus vaccines), which induces cross-linking of proteins, γ-rays have little impact on the antigenic structure and biological integrity of proteins [7]. The Manual on Radiation Sterilization of Medical and Biological Material of the International Atomic Energy Agency indicates that exposure to 0.65 kGy of γ-rays causes a total loss of influenza virus infectivity, but disrupting the haemagglutinating activity requires an exposure to higher than 200 kGy [9]. The reduced impact of γ-irradiation on the antigenic structure of viral particles is therefore also expected to improve the magnitude and/or quality of humoral immunity elicited in vaccine recipients over that obtained by using present vaccine preparations. We have been investigating the ability of γ-Flu preparations to induce heterotypic and cross-protective immunity against avian H5N1 influenza A virus. Our data clearly show that a single intranasal administration of γ-A/PR8[H1N1] protects mice against lethal H5N1 and other heterotypic infections.

## Materials and Methods

### Mice

Nine- to ten-week-old female BALB/c mice were routinely used in these studies. For H1 and H3 experiments, mice were obtained and housed in Biosecurity Level 2 containment facilities at the John Curtin School of Medical Research, the Australian National University, ACT, Australia. For H5 studies, which were conducted at the Australian Animal Health Laboratory (AAHL; Geelong, Australia), mice were obtained from the Animal Resource Centre (Perth, Australia), and all work using live virus was carried out under Biosecurity Level 3 enhanced containment. All experimental procedures were approved by the institutional Animal Ethics Committees.

### Viruses and cells

P815 and Madin-Darby canine kidney (MDCK) cells were maintained in F15 plus 5% foetal calf serum (FCS) and incubated at 37°C in a humidified atmosphere with 5% CO_2_.

Stocks of influenza A viruses, (A/PR8 (A/Puerto Rico/8/34 [H1N1]), A/PC (A/Port Chambers/1/73 [H3N2]), A/JAP (A/Japan/305/57 [H2N2]), and A/Vietnam/1203/2004[H5N1]), were grown in embryonated hen eggs. Virus stocks were prepared from allantoic fluid and stored in aliquots at −70°C. A/Vietnam/1203/2004 was obtained from the WHO Collaborating Centre for Reference and Research on Influenza (Melbourne, Australia).

### Virus titration

Virus content for A/PR8[H1N1], A/PC[H3N2], and A/JAP[H2N2] stocks were determined by standard plaque assay on MDCK cells. Virus titres for these stocks were 8×10^7^ plaque forming unit (PFU)/ml, 1×10^7^ PFU/ml, and 1×10^7^ PFU/ml, respectively.

Titration of H5N1 infectivity was routinely undertaken by infection of replicate Vero cell monolayers with 10-fold serial dilutions of sample in Eagle's minimum essential medium with Earle's salts (EMEM) containing 10% FCS and antibiotics. Plates were incubated at 37°C for 5 days in 5% CO_2_, and the number of replicate wells in each dilution series with cytopathic effect determined. Titration of stock virus in 13-week-old female BALB/c mice was performed by intranasal inoculation of groups of 5 mice with 35 µl of 10-fold serially diluted virus in phosphate buffered saline (PBS). Mice were monitored for development of signs consistent with influenza infection for 10 days and were euthanised if their clinical status met any of the following criteria: loss of 20% of the pre-challenge body weight, development of any neurological sign or an inability to eat or drink. Both 50% tissue culture infectious dose (TCID_50_) and 50% mouse infectious dose (MID_50_) titres were calculated using the method of Reed and Muench [10]. For the stock H5N1 virus, the titre was 10^9.0^ TCID_50_/ml or 10^7.0^ MID_50_/ml.

### γ-Flu preparations

Stocks of A/PR8[H1N1] and A/PC[H3N2] were purified by temperature-dependent adsorption to chicken red blood cells (CRBC) [11]. Briefly, infectious allantoic fluids were incubated with CRBC for 45 min at 4°C. Then, stocks were centrifuged at 1200×g for 10 min and supernatants discarded. Pellets (CRBC and attached viruses) were resuspended in normal saline and incubated for 1 h at 37°C. Following incubation, samples were centrifuged (1200×g for 10 min), the supernatants collected and virus titres estimated by plaque assays on MDCK cells. The purified stocks were stored at −70°C, inactivated by exposure to 10 kGy of γ-irradiation (Australian Nuclear Science and Technology Organization – ANSTO, Lucas Heights, Australia), and tested for residual infectivity using embryonated hen eggs. These stocks were sterile but retained full haemagglutinating activity after irradiation.

### ^51^Cr-release cytotoxicity assay

A/PR8, A/PC or their corresponding γ-Flu preparations (2×10^7^ PFU equivalents per mouse) were administered intravenously to 10-week-old BALB/c mice. Six days later, splenocytes from infected, vaccinated, or mock-immunized animals were harvested and tested for their killing activity on mock, A/PC-, A/PR8- or A/JAP[H2N2]-infected target cells or labeled with the K^d^ restricted nucleoprotein derived peptide TYQRTRALV (NPP) using Cr^51^ release assays, as previously described [12]. Red blood cell depleted splenocytes (effectors) were mixed with labeled targets at different ratios and incubated for 8 h at 37°C in a humidified atmosphere containing 5% CO_2_. The level of radioactivity in supernatants was measured and the specific lysis calculated using the formula: (experimental cpm – spontaneous cpm)/(maximal release cpm – spontaneous cpm)×100.

### Vaccination and viral challenge

Mice were anaesthetised by intraperitoneal administration of ketamine HCl (100 mg/kg) and xylazine HCl (10 mg/kg) prior to vaccination or challenge. Mice were vaccinated by intranasal administration of 32 µl (3.2×10^6^ PFU equivalents/mouse) of γ-Flu (γ-A/PR8 or γ-A/PC) divided equally between both nostrils. Mock vaccinated mice received diluent alone (PBS). For H1 and H3 studies, four weeks following vaccination, animals were challenged intranasally with 6×10^2^ PFU/mouse of live virus (A/PR8[H1N1] or A/PC[H3N2]), and mice were weighed prior to infection and then daily for a period of 21 days.

For H5 studies, four weeks following vaccination, mice were challenged with 35 µl (3 MID_50_) of H5N1 virus, divided equally between nostrils. Two or three mice from each group were euthanised on day 3 and 6 post-challenge, respectively, and right lung and brain were collected for determination of viral genetic load and infectivity, using separate sterile instruments for every tissue to prevent cross contamination between samples. Tissues were diced using separate sterile disposable scalpels and stored in PBS, on ice, until transferred to −70°C for longer term storage. The remaining 10 mice in each group were weighed daily (twice daily once 15% body weight loss was detected), examined twice daily for signs consistent with H5N1 infection, and euthanised according to the experimental endpoints described earlier for determination of MID_50_.

### Homogenisation of tissues

Lung and brain homogenates were generated in 1 ml of PBS using a Mini-BeadBeater-8 (Biospec Products, USA), and adjusted to 10% (w/v) in PBS prior to extraction of viral RNA or titration of infectivity.

### Real-time RT PCR

Following the addition of 100 µl of lung or brain homogenate into 600 µl of RLT buffer, RNA was extracted using an RNeasy Mini Kit (Qiagen) according to the manufacturer's instructions. The total RNA concentration of each sample was determined by spectrophotometry and adjusted to 40 ng/µl with nuclease free water. Standardised amounts (200 ng) of template were subsequently reverse transcribed using the TaqMan Reverse Transcription Reagents kit (Applied Biosystems) in 20 µl reactions following the recommendations of the manufacturer. For quantitation of viral cDNA, universal influenza virus type A-specific primers and TaqMan probe, which amplified and detected a product from within the viral matrix gene, were used [13]. Reactions were performed in triplicate and contained 12.5 µl of TaqMan 2× Universal PCR Master Mix, 900 nM of each primer, 250 nM of probe, 2 µl of cDNA template and 6.8 µl of water. Separate triplicate reactions to quantify 18S rRNA (TaqMan Ribosomal Control Reagents, Applied Biosystems) were also performed to exclude the presence of PCR inhibitors in all samples tested. Reactions were performed in 96-well plates using the 7500 Fast Real-Time PCR System (Applied Biosystems) and the following cycling parameters: 50°C for 2 min; 95°C for 10 min; 45 cycles of 95°C for 15 sec and 60°C for 1 min. For relative quantitation of viral genetic loads, a standard curve was generated using, as template, 10-fold serial dilutions of extracted stock virus RNA in 40 ng/µl of RNA prepared from uninfected mouse lung. To facilitate interpretation of data, 1 unit (1 U) of viral RNA was arbitrarily defined as the number of RNA molecules which, when reverse transcribed and subjected to real-time PCR, produced a C_T_ value of 38.

## Results

### Gamma-Flu preparations induce cross-reactive cytotoxic T cell responses

Virus stocks of two influenza A virus strains (A/PR8[H1N1] and A/PC[H3N2]) were grown in embryonated hen eggs, purified by temperature-dependent adsorption to chicken red blood cells [11] and titrated by plaque assay on MDCK cells. The purified stocks were exposed to 10 kGy of γ-irradiation, and tested for residual viral infectivity by using embryonated hen eggs and plaque assay on MDCK cells. Virus stocks were sterile but retained full haemagglutinating activity.

**Figure 1:**
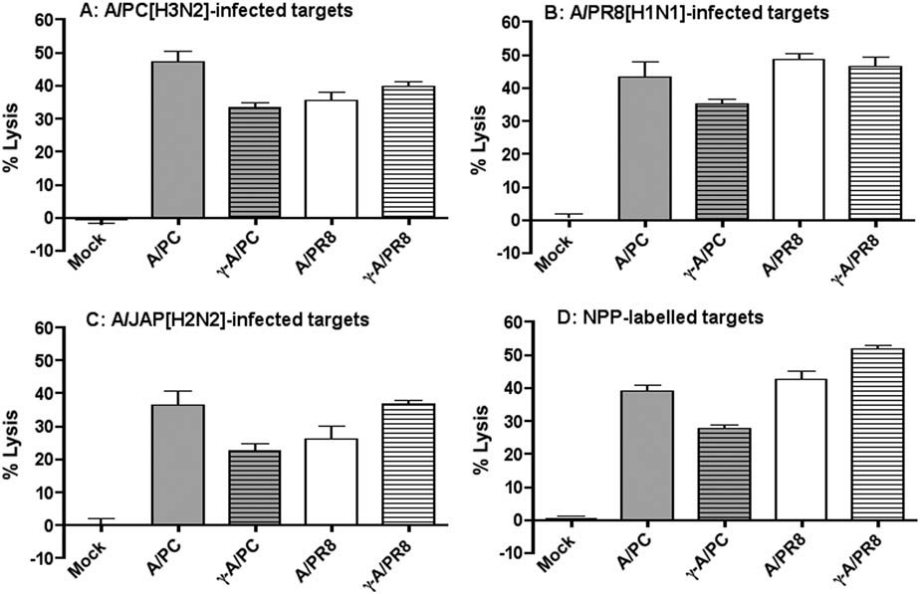
Cross-reactive cytotoxic T cell responses induced by γ-Flu. 10-week-old BALB/c mice were either infected or vaccinated with live A/PR8, γ-A/PR8, live A/PC, or γ-A/PC. Six days later, splenocytes from these mice were tested for their killing activity against mock, A/PC-, A/PR8-, A/JAP-infected, and NPP-labelled P815 targets. Data represent % specific lysis after 6 h assay time at an effector to target cell ratio of 120∶1.

To test the ability of γ-Flu to induce cross-reactive Tc cell responses A/PR8, A/PC and their corresponding γ-Flu preparations were used to infect or vaccinate mice. Six days later splenocytes from infected, vaccinated, and mock-immunized animals were tested for their killing activity on mock, A/PC-, A/PR8-, A/JAP[H2N2]-infected or H-2K^d^ restricted nucleoprotein-derived peptide (NPP)-labeled P815 target cells using a standard ^51^Cr release cytotoxicity assay [12]. All effector splenocytes from flu-infected and γ-Flu-vaccinated animals expressed lytic activity against all influenza infected P815 targets regardless of the virus strains used (Figure 1). In addition, all splenocyte populations, except those from mock-infected mice, killed NPP-labeled targets. Thus, γ-Flu preparations induce cross-reactive influenza-immune Tc cell responses in mice.

### Routes of vaccination

**Figure 2:**
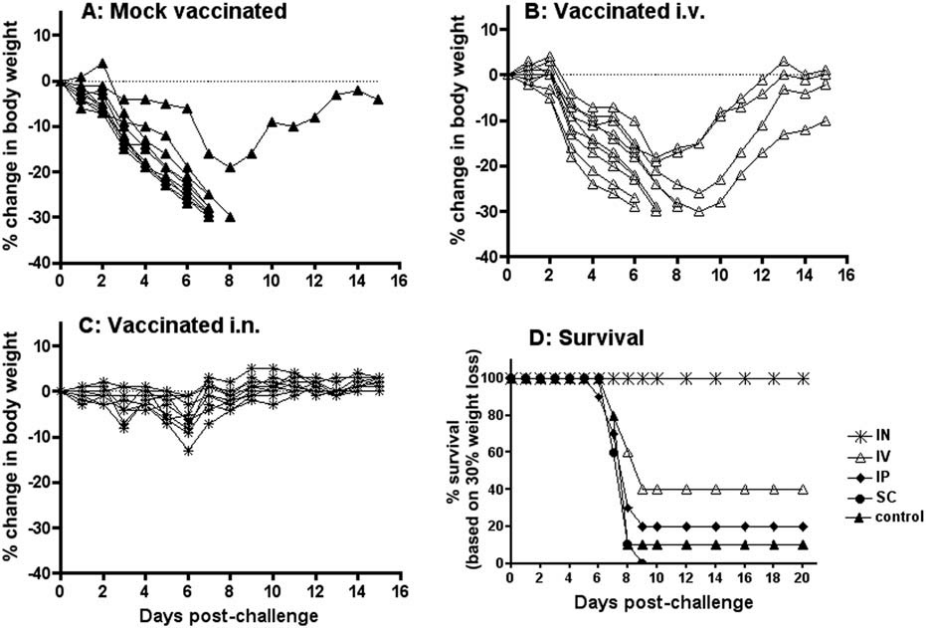
Intranasal vaccination with γ−Flu provides superior protection to heterotypic virus challenge. Groups of 10 BALB/c mice were either mock treated (A) or vaccinated with γ−A/PC (3.2×10^6^ PFU equivalents) intravenously (B) or intranasally (C). Mice were challenged intranasally after 3 weeks with a lethal dose (6×10^2^ PFU) of A/PR8 and weight recorded daily for 21 days. Survival (D) of mice mock treated, or vaccinated i.n., i.v., i.p., or s.c. and challenged as for (A–C) and monitored for 21 days.

Different routes of vaccination (intranasal (i.n.), intravenous (i.v.), intraperitoneal (i.p.) and subcutaneous (s.c.)) were used to immunize BALB/c mice (10 mice/group) with 3.2×10^6^ PFU equivalents of γ-Flu (γ-A/PC[H3N2]). Three weeks post vaccination mice were challenged i.n. with a lethal dose of live A/PR8[H1N1] (6×10^2^ PFU) and monitored for mortality and clinical signs using 30% body weight loss as the end point (Figure 2). All i.n. vaccinated animals fully recovered with little, if any, weight loss after challenge with the heterotypic virus (Figure 2C). In contrast, the majority of mock vaccinated (Figure 2A), i.v. vaccinated (Figure 2B), and i.p. and s.c. vaccinated (data not shown) mice lost weight progressively to reach 30% body weight loss by days 7 or 8 post-challenge. The survival data (Figure 2D) show that despite the use of high challenge doses of A/PR8[H1N1], i.n. vaccinated mice survived the heterotypic challenge at significant levels (P<0.05 using Fisher's Exact test). In addition, mice vaccinated with γ-A/PC or γ-A/PR8 survived both heterotypic and homotypic challenges with 50× the lethal dose of A/PR8 (data not shown). Thus, γ-Flu represents a new vaccine concept that induces highly efficient heterotypic protection against influenza A strains.

### Intranasal vaccination with γ-A/PR8[H1N1] protects against lethal H5N1 infection

**Figure 3:**
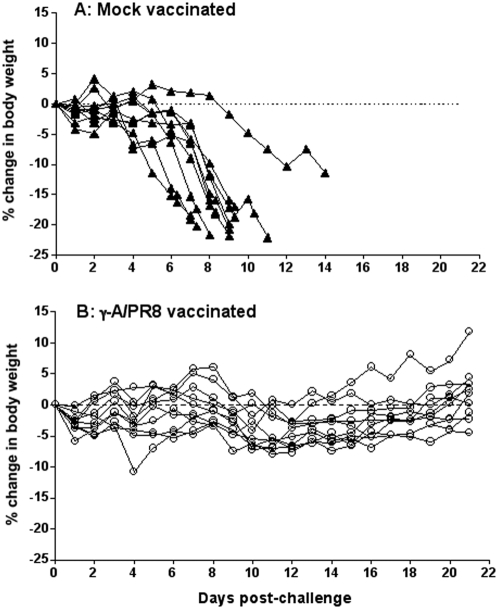
Intranasal vaccination with γ−Flu (γ−A/PR8[H1N1]) protects against H5N1 challenge. Groups of 10 BALB/c mice were either mock treated (A) or vaccinated with γ−A/PR8 (B). Mice were challenged 4 weeks later with 3 MID_50_ of A/Vietnam/1203/2004[H5N1] intranasally and weight recorded daily for 21 days.

To determine the extent to which the heterotypic immunity induced by γ-Flu preparations of human influenza A viruses extends to avian isolates, we tested the protective efficacy of γ-A/PR8[H1N1] using a mouse model of H5N1 highly pathogenic avian influenza. BALB/c mice (15 mice/group) were vaccinated i.n. with a single dose of γ-A/PR8[H1N1] (3.2×10^6^ PFU equivalents/mouse). Four weeks later, under Biosecurity Level 3 enhanced containment, mice were challenged i.n. with 3× the 50% mouse infectious dose (3 MID_50_) of A/Vietnam/1203/2004[H5N1]. Two or three mice from each group were euthanised on day 3 and 6 post-challenge, respectively, for determination of viral genetic load and infectivity in lung and brain, while the remaining 10 mice in each group were monitored for development of clinical signs and loss of body weight (Figure 3). All ten mice in the mock vaccinated group developed clinical signs consistent with H5N1 infection and were euthanised between days 7 and 14 post-challenge in accordance with the experimental end points that were approved by the institutional animal ethics committees (weight loss of 20%, development of any neurological sign, or inability to eat or drink) (Figure 3A). Mock vaccinated mice developed greasy, ruffled fur from day 4 post-challenge, which progressively worsened until euthanised. Two mice developed neurological signs categorized by an abnormal hind limb gait and hind limb weakness, at which time each was euthanised (day 9 or 14 post-challenge), while all other mice were euthanised at ∼17–22% body weight loss with varying degrees of depression, inactivity and dehydration. In contrast, all vaccinated mice (γ-A/PR8[H1N1]) remained bright and active throughout the study and were euthanised at the conclusion of the trial on day 21 post-challenge (Figure 3B). Although one mouse had lost ∼11% body weight by day 4 post-challenge, it was otherwise bright and active, and had regained its pre-challenge weight by the end of the trial. Furthermore, despite most mice having lost up to ∼7–8% body weight from day 6 through to 12 post-challenge, all gained weight thereafter such that all but three had reached or exceeded their pre-challenge weight by day 21 post-challenge. Therefore, our data demonstrate that a single dose of γ-Flu administered i.n. induces cross-protective immunity in mice against a lethal challenge with H5N1 virus. Quantitation of viral infectivity and viral genetic loads in lung and brain confirmed the protective effect of γ-A/PR8[H1N1] against avian influenza and demonstrated clearance of H5N1 virus from lung tissues by day 6 post-challenge (Table 1). The detection of viral infectivity and viral RNA in the lung of 1 vaccinated mouse on day 3 post-challenge but not on day 6 indicates that the observed cross-protective immunity is most likely mediated by memory Tc cells, which express accelerated activation kinetics over that of naïve Tc cells [14]. We also have preliminary data showing that passive transfer of CD8^+^ T cells, but not serum, from mice vaccinated 6 days previously with γ-Flu can confer protection against lethal heterotypic infection (Furuya et al, in preparation).

**Table 1:** H5N1 infectivity and viral genetic loads in lung and brain.

Day*	Treatment	Mouse	Infectivity		Genetic load	
			Lung	Brain	Lung	Brain
	Mock	1	6.5	≤3.2	3.4±0.03	-†
3		2	5.8	-	2.0±0.02	-
	Vaccinated	1	-	-	-	-
		2	7.4	-	3.6±0.02	-
	Mock	1	6.4	-	4.2±0.003	-
6		2	6.4	-	4.2±0.01	-
		3	7.4	5.0	4.4±0.03	3.0±0.03
	Vaccinated	1	-	-	-	-
		2	-	-	-	-
		3	-	-	-	-

NOTE. Viral infectivity and relative viral genetic loads are expressed as log_10_ TCID_50_/g and log_10_ U per 20 ng of extracted RNA (geometric mean±s.d. of triplicate reactions), respectively, where 1 unit (1 U) of viral RNA is arbitrarily defined as the number of RNA molecules which, when reverse transcribed and subjected to real-time PCR, produced a C_T_ value of 38.

* Day post-challenge.

† Undetectable (<10^3.2^ TCID_50_/g (infectivity) or <1 U per 20 ng of extracted RNA (genetic load)).

## Discussion

Human influenza A viruses bind to α2,6-linked sialic acid receptors expressed on epithelial cells of the upper respiratory tract of humans, whereas avian influenza H5N1 viruses bind to α2,3-linked receptors expressed predominantly in the lower respiratory tract [15], [16] In general, binding of virus to α2,6-linked receptors is associated with low virulence but high transmissibility. In contrast, virus binding to α2,3-linked receptors is associated with low transmissibility but high virulence and often lethal influenza virus infections [16]. Therefore, an avian influenza pandemic might be expected to be associated with a mutation in the haemagglutinin molecule that would allow H5N1 virus to bind to α2,6-linked receptors. Subunit vaccines based on currently identified H5 molecules cannot account for the antigenic variants that would result following such mutations and, consequently, neutralizing antibody-mediated protection induced by these vaccines would likely be limited. Tc cell responses to influenza infection, however, are mainly directed against internal viral proteins, which are highly conserved among all influenza A virus strains [4]. Mutations of internal genes are not susceptible to antibody-mediated selection and viral escape from Tc cell responses is curtailed by MHC class I polymorphism. Therefore, vaccines inducing Tc cell responses against the highly conserved internal proteins, such as the nucleoprotein, are expected to provide cross-protection against different influenza A virus strains [17], [18]. Our data indicate that the cross-reactive Tc cell responses induced by γ-Flu are predominantly directed against the internal proteins, indicated by lysis of NPP-labeled targets (Figure 1D).

The Tc cell response is broadly cross-reactive between influenza A strains and is important for recovery from primary infections in combination with antibodies [19], [20] Furthermore, cross-recognition of avian H5N1 influenza virus by human Tc-lymphocyte populations induced by human influenza A virus has recently been reported [21]. We have previously discussed the potential applicability of γ-Flu as vaccine candidates to induce cross-protective immunity and envisaged two, not exclusive, mechanisms to induce efficient Tc cell responses: efficient cellular uptake of γ-Flu and abortive translation of fragmented genomes [22]. While the abortive translation represents a very remote possibility due to the negative strandedness of the influenza virus genome, an efficient cellular uptake of γ-irradiated virus particles is the most likely mechanism to the induction of Tc cell responses. Nonetheless, the ability to induce Tc cell responses should be considered a highly desirable property of an inactivated influenza virus vaccine candidate. This, however, does not exclude the importance of antibodies, including the possibility of an enhanced cross-protective antibody response. The underlying mechanisms for the cross-protective immunity are currently under investigation, and our preliminary data strongly point to a Tc cell mediated mechanism. This is based on the following observations. One, passive transfer of T cells, but not B cells or serum, from immunised mice protects naïve mice from heterotypic influenza infections. Two, MHC class I deficient mice (beta-2 microglobulin knock out) are not protected against heterotypic challenge and finally perforin deficient mice are not protected when gamma-flu immunized mice are challenged with lethal doses of live heterotypic viruses (Furuya et al, manuscript in preparation). In addition it is of interest and supports our contention of a Tc cell mediated mechanism that gamma-flu like live virus induces Tc cell responses while other virus inactivation procedures (formalin or UV inactivation) as well as commercially available flu vaccines are unable to do so (Furuya et al submitted).

Given the importance of obtaining improved heterotypic immunity and protection, our novel vaccine concept may overcome the poor efficacy of present influenza vaccines against antigenic variants arising following host range mutations in virulent avian or porcine strains. Therefore, γ-Flu represents a novel vaccine concept, with the potential to protect not only against seasonal flu infections but also possible avian flu pandemics. Unlike experimentally attenuated (cold adapted) viruses, γ-Flu is unable to revert to live virulent virus. We do not yet fully understand why γ-Flu is eliciting immunity similar to that obtained with live virus. However, the observation that γ-Flu, unlike other γ-ray inactivated viruses [23], induces a vigorous Type I interferon response with an accompanying partial systemic lymphocyte activation (Furuya et al, unpublished data) may be at least partially responsible for its superior immunogenicity. Furthermore, intranasal vaccination would make administration of such a vaccine preparation highly advantageous in developing countries and high priority should be given to evaluating its efficacy in humans.
